# Preliminary Study on the Functions of Peptides Obtained from White Mullet (*Ophiocephalus argus* var. Kimnra) Meat

**DOI:** 10.3390/foods14081322

**Published:** 2025-04-11

**Authors:** Yin Zhang, Pengcheng Zhang, Qiuyue Chen, Aodong Wang, Li Dong, Longyi Zhang

**Affiliations:** Meat Processing Key Laboratory of Sichuan Province, Chengdu University, Chengdu 610106, China

**Keywords:** white mullet, peptides, antioxidation, functional peptides, lactation function

## Abstract

To explore the functions of peptides obtained from white mullet (*Ophiocephalus argus* var. Kimnra) meat, the meat was hydrolyzed via simulated digestion in vitro, and the functions (milk secretion ability, antioxidant activity, angiotensin-converting enzyme (ACE) inhibitory activity, and Fe^2+^ chelation) of the obtained peptide were evaluated. The results indicated that both low-dose and high-dose peptide promoted milk secretion in lactating rats in vivo; the peptides had scavenging effects on free radicals of 1,1-diphenyl-2-trinitrophenylhydrazine (DPPH), 2,2′-azino-bis (3-ethylbenzo-thiazoline-6-sulfonic acid) diammonium salt (ABTS), OH^−^, and O^2−^, and the EC_50_ concentrations were 55.94 mg/mL, 10.14 mg/mL, 52.92 mg/mL, and 28.53 mg/mL, respectively. The peptides had an inhibitory effect on ACE, and the IC_50_ concentration was 15.81 mg/mL. The peptides had a chelating ability to Fe^2+^, and the IC_50_ concentration was 69.05 mg/mL. These results indicate that peptides obtained from white mullet meat exhibit milk secretion-promoting ability, antioxidant activity, ACE-inhibitory activity, and Fe^2+^ chelation, making this an effective approach for isolating specific functional peptides and identifying their sequences from the digested solution of white mullet meat.

## 1. Introduction

White mullet (*Ophiocephalus argus* var. Kimnra) is a cultivated freshwater fish species with a breeding area in Sichuan Province of approximately 3.87 square kilometers, an output of more than 520 tons, and a comprehensive output value of approximately CNY 120 million [1]. The white mullet is different from black fish (*Channa argus* [2]) in that it has a white body color, a flat head, larger scales on the head, and thinner scales on the tail, while black fish has a black body color, a wider head, and smaller scales. The white mullet meat is characterized by a delicious taste, tender texture, and fewer bones [3]. It has been found that white mullet meat has the functions of promoting milk secretion in postpartum women and wound healing in postoperative patients [4]. However, there is very little research on these functions. Peptides isolated from meat using hydrolysis have shown antioxidant, anti-inflammatory, antibacterial, anti-fatigue, anticancer, hypotensive, and metal-ion-chelating effects [5,6]. Lan et al. [7] obtained the peptide GMKCAF, which has an angiotensin-converting enzyme (ACE)-inhibitory effect, from lizard fish via neutral protease. Wu et al. [8] identified two antimicrobial peptides (FLKSIWRAAKGAIRGAKSGWRA and FFGMLIHGAI) from Japanese seabass (*Lateolabrax japonicus*), while Hashem et al. [9] identified two antioxidant peptides (HNLGLLHGDM and DAPSMND) from *Piaractus brachypomus* meat hydrolysate. These investigations suggest that the peptides found in white mullet meat might contribute to its beneficial functions. To confirm this hypothesis, in this study, white mullet meat was hydrolyzed to obtain peptides and their functions were evaluated.

The most commonly used method for obtaining peptides from meat and its byproducts is enzymatic hydrolysis through protease treatment. The enzymes frequently used are papain, alkaline protease, neutral protease, pepsin, trypsin, and flavourzyme [10,11,12,13,14]. Yin [15] isolated antioxidant active peptides from grass carp (*Ctenopharyngodon idella*) meat using papain and neutral protease, and Wang [16] used alkaline protease to hydrolyze grass carp (*Ctenopharyngodon idella*) meat to obtain ACE-inhibitory peptides. Zamora-Sillero et al. [17] reviewed the functional peptides obtained from fish by-products using protease hydrolysates. However, the peptides obtained using enzymatic hydrolysis are not an appropriate substitute for peptides generated during human digestion; thus, they cannot be used to prove that eating white mullet meat is conductive to the promotion of milk secretion or wound healing. It also limits the discovery of other functionalities of peptides from white mullet meat after being digested by the human stomach.

In order to obtain direct evidence to prove the functionality of white mullet meat after digestion and verify that the peptides produced from digestion are functional, the method of simulated digestion in vitro was adopted to hydrolyze the white mullet meat. This method has been widely used in evaluating the dietary intake of bioactive substances [18]; for example, it has been used to compare the effect of frying and boiling on the nutritional value of rabbit meat [19], and to obtain antioxidant peptides from the cooked juice of crucian carp (*Carassius auratus*) meat [20]. Based on these investigations, a white mullet meat peptide was obtained through simulated digestion in vitro and its functions (milk secretion ability, antioxidant activity, ACE-inhibitory activity, and Fe^2+^ chelation ability) were evaluated. The results of this investigation can not only be used to prove the functionalities of peptides obtained from white mullet meat after digestion, but are also beneficial in the identification of new functional peptides in white mullet meat. Moreover, it helps to gain a deeper understanding of the nutritional value and health benefits of the white mullet meat protein, and also provides new ideas and directions for the innovation and development in the seafood processing industry.

## 2. Materials and Methods

### 2.1. Materials and Reagents

The white mullet was sent to our laboratory from a white mullet breeding base in Yong’an Town (Neijiang, China). Pregnant SD rats were bought from Chengdu Dashuo Animal Co., Ltd. (Chengdu, China). Pepsin (EC 3.4.23.1, 10,000 U/g), trypsin (EC 3.4.21.4, 2500 U/g), 1,1-diphenyl-2-trinitrophenylhydrazine (DPPH·), 2,2-azino-bis (3-ethylbenzothiazoline-6-sulfonic acid) diammonium salt (ABTS), nitrotetrazolium chloride blue (NBT), reducing coenzyme I disodium salt (NADH), phenazine N-methyl sulfate, phenanthroline, 1, 10-phenanthroline, hippuroyl–histidine–leucine (HHL), angiotensin-converting enzyme (ACE, EC 3.4.15.1, ≥2.0 units/mg), and chloral hydrate were purchased from Shanghai Yuanye Biotechnology Co., Ltd. (Shanghai, China). Sodium bicarbonate, sodium hydroxide, anhydrous ethanol, and ferrous sulfate were purchased from Chengdu Cologne Chemicals Co., Ltd. (Chengdu, China). Potassium persulfate was purchased from Tianjin Zhiyuan Chemical Reagent Co., Ltd. (Tianjin, China). Ferrous chloride was purchased from Tianjin Dongtianzheng Fine Chemical Reagent Factory (Tianjin, China). Ethylenediamine tetraacetic acid was bought from Chengdu Jinshan Chemical Reagent Co., Ltd. (Chengdu, China), and dipotassium hydrogen phosphate and potassium dihydrogen phosphate were purchased from Tianjin Kemiou Chemical Reagent Co., Ltd. (Tianjin, China). A rat prolactin (PRL) quantitative detection kit and a rat 5-hydroxytryptamine (5-HT) quantitative detection kit were purchased from Quanzhou Ruixin Biotechnology Co., Ltd. (Quanzhou, China).

### 2.2. Sample Pretreatment

The white mullet with a weight of 500.0 ± 50.0 g was transported to our laboratory within 3 h under temporary breeding and treated according to the method described by Zhang et al. [21]. In accordance with the decision made by the Institutional Animal Care and Use Committee of Chengdu University, the fish used in the experiments were washed with tap-water, dried with filter paper, and killed by knocking the head. The fish meat from the belly was sampled, put into a sealed bag, and stored at 4 °C for later use.

### 2.3. Preparation of Peptide from White Mullet Meat

The INFOGEST [22] in vitro digestion protocol is mainly used for the in vitro digestion of starch, fat, and protein in food, which is inconsistent with the preparation of digested peptide from the white mullet meat protein in this study. To prepare the digested peptides, a protein digestion model was used [20]. The white mullet meat was chopped into 5 mm pieces and mixed with pure water at a ratio of 1:4 (*w*/*v*). A SCIENTZ-11 type homogenizer (Ningbo Xinzhi Biotechnology Co., Ltd., Ningbo, China) was adopted to homogenize the meat for 2 min at room temperature (25 ± 2 °C) to form a white mullet meat mixture. The meat mixture was treated using the simulated digestion method described by Zhang, Du, Zhang, Kong, Hu, Xiong, and Zhao [20] with slight modifications. The mixture was adjusted to pH 2.0 with 1 mol/L HCl. Based on meat weight, pepsin (4% of the weight of meat) was added, and gastric digestion was simulated for 2.5 h at 37 °C in a HH-S6 thermostat water bath (Beijing Kewei Yongxing Instrument Co., Ltd., Beijing, China). The mixed solution was adjusted to pH 5.33 with 0.9 mol/L NaHCO_3_ and pH 7.5 with 1 mol/L NaOH. Based on meat weight, trypsin (4% of the weight of meat) was added and intestinal digestion was simulated at 37 °C in a HH-S6 thermostat water bath for 2.5 h. The solution was then heated in a water bath (>90 °C) for 10 min to terminate the digestion. Then, the mixture was cooled to room temperature (25 ± 2 °C) and centrifuged at 8000× *g* and 4 °C for 20 min using a TLG-1650 refrigerated centrifuge (Sichuan Shuke Instrument Co., Ltd., Chengdu, China) to obtain the supernatant. The supernatant was freeze-dried using an FD-1A-80 vacuum freeze-dryer (Beijing Bo medical Kang technology Co., Ltd., Beijing, China), referring to the method of Zhang et al. [23] with slight modifications, and the freeze-dried peptide powder was obtained.

### 2.4. Determination of Galactagogue Activity

#### 2.4.1. Establishment of Animal Model

SD pregnant rats with a labor period of about 1 week were transported from Chengdu Dashuo Animal Co., Ltd. to the animal house in Chengdu University for temporary breeding. The animal model was established according to Guo et al. [24] with slight modifications. The number of rats used in each group was determined based on Lin et al. [25]. Thirty female rats with a difference of less than 24 h in litter time were selected as the experimental research objects and divided into the following groups: normal (NL), model (MD), low-dose peptide (LP), high-dose peptide (HP), and white mullet meat (WM), with 6 pregnant rats in each group. Except for those in the NL group, the rats in each group were intragastrically administered 1.6 mg/kg bromohintine each day following the second day of delivery. The rats in the NL group were intragastrically administered 20 mg/mL of distilled water every day, and those in the LP group were given 20 mg/mL freeze-dried peptide solution (low-dose) and fed ordinary feed every day. The rats in the HP group were intragastrically administered 50 mg/mL freeze-dried peptide solution (high-dose) and fed ordinary feed every day. From the second day of delivery, the NL and MD rats were fed daily with distilled water and ordinary feed, while the WM group rats were fed daily with distilled water and feed containing white mullet meat. The ordinary feed was bought from Chengdu Dashuo Animal Co., Ltd. (Chengdu, China). The feed containing white mullet was customized at Chengdu Dashuo Animal Co., Ltd. It was prepared by adding 7% (*w*/*w*) white mullet meat into the ordinary feed and was formed and dried using the same process as the ordinary feed.

#### 2.4.2. Lactation Volume of Rats

The lactation volume of the rats was determined according to the daily change in body weight of the whole litter of rat pups, referring to the method of Lin, Wang, Yao, Zhong, Lin, and You [25] with some modifications. The whole litter of rat pups (15 pups) was weighed (W_1_) and separated from the postpartum rats at 1:00 PM. The first separation period lasted 4 h and ensured that the pups did not have the opportunity to suckle. During this time, the postpartum rats were taken from the cage and placed in a separate clean cage. This period was designed to starve the pups. Next, the pups were weighed (W_2_) at 5:00 PM. The average weight change rate within 4 h was used as a basal metabolic rate. Following this, the pups were returned to the postpartum rats’ cage for 1 h for suckling. In theory, the total amount of milk suckled by the pups is regarded as the level of milk produced from the mammary glands of postpartum mice. At the end of the 1 h suckling period, the weight of the whole litter of rat pups was recorded (W_3_). Milk production was calculated as the difference between the final and initial pup weights. Considering the basal metabolic rate of the pups during the 1 h nursing period, the lactation yield (LY) was calculated according to Formula (1):

LY (g/h) = (W_3_ − W_2_)/1 + (W_1_ − W_2_)/4 (1)

The lactation yield was continuously recorded for 13 days.

#### 2.4.3. Net Weight Gain

The net weight gain (NWG) was calculated according to the method described by Lin, Wang, Yao, Zhong, Lin, and You [25]. The net weight gain was the weight gained by the rat pups in each litter for 13 days, and was calculated according to Formula (2):

NWG = W_4_ − W_0_ (2)

where W_4_ is the weight per litter of rat pups on the last day, and W_0_ is the weight per litter on the first day.

#### 2.4.4. Determination of Prolactin (PRL) and 5-Hydroxytryptamine (5-HT)

At the end of the feeding experiment, in accordance with the decision made by the Institutional Animal Care and Use Committee of Chengdu University, the rats in each group were injected intraperitoneally with 1 mL 10% chloral hydrate for anesthesia, and then blood was sampled via enophthalmectomy. After standing at normal temperature (25 ± 2 °C) for 30 min, the serum was separated using a TLG-1650 centrifuge machine at 3000 r/min and 4 °C for 15 min. A rat PRL quantitative detection kit (ELISA, Quanzhou Ruixin Biotechnology Co., Ltd., Quanzhou, China) and a rat 5-HT quantitative detection kit (ELISA, Quanzhou Ruixin Biotechnology Co., Ltd., Quanzhou, China) were used for determining the content of PRL and 5-HT according to the operating instructions of the kit.

#### 2.4.5. Hematoxylin–Eosin (HE) Staining of Breast Histology

After blood collection under anesthesia, and in accordance with the decision made by the Animal Ethics Committee of Chengdu University, the rats were dissected immediately, and tissue was taken from the fourth set of mammary glands and fixed with 4% paraformaldehyde for 24 h. After dehydration, the tissue was embedded in paraffin wax and then sliced using a paraffin microtome (thickness 3 μm). Finally, the pathological changes in the breast tissue were observed with a Pannoramic MIDI II digital slice scanner (Shanghai Damai Biotechnology Co., Ltd., Shanghai, China) after hematoxylin–eosin staining.

### 2.5. Determination of Antioxidant Activity

#### 2.5.1. DPPH· Radical Scavenging Rate

The DPPH· free radical clearance rate of peptides was determined according to the national standard GB/T 39100-2020 of China [26] with slight modifications. Briefly, 5 mg of DPPH· was dissolved into 100 mL anhydrous ethanol and then stored away from light. For the test group (A_1_), 0.3 mL of DPPH· anhydrous ethanol solution was added to 0.1 mL peptide solution with different concentrations; for the control group (A_2_), 0.3 mL anhydrous ethanol was added to 0.1 mL white mullet meat peptide solution with different concentrations; and for the blank group (A_0_), 0.3 mL of DPPH· anhydrous ethanol solution was added to 0.1 mL pure water. After being fully mixed, the absorbance was measured at 517 nm for 30 min with a Sky High microcoder (Thermo Fisher (Shanghai) Instrument Co., Ltd., Shanghai, China), and the clearance rate (CR) was calculated according to Formula (3). Taking the concentration of the sample solution as the horizontal coordinate and the clearance rate as the vertical coordinate, the linear equation between the sample solution and clearance rate was established to calculate the concentration of half clearance EC_50_. Each sample was evaluated in triplicate.

CR (%) = (1 − (A_1_ − A_2_)/A_0_) × 100% (3)

#### 2.5.2. ABTS· Radical Scavenging Rate

The ABTS· free radical clearance rate (RCR) of the peptide was determined according to the national standard GB/T 39100-2020 of China [26] with slight modifications. To make the ABTS· concentrated solution, 200 mg of ABTS· and 34.4 mg of potassium persulfate were dissolved into 50 mL deionized water at room temperature (25 ± 2 °C) for 24 h away from light. Then, the ABTS· concentrated liquor was diluted with 95% ethanol to an absorbance of 0.70 ± 0.02 (OD value: 734). To create the test group (A_1_), 0.1 mL white mullet meat peptide solution of different concentrations was mixed with 0.9 mL ABTS· diluent solution, and for the blank group (A_0_), 0.1 mL pure water was added to 0.9 mL ABTS· diluent solution. After fully mixing the samples, the absorbance was measured at a 734 nm wavelength after reflection at room temperature (25 ± 2 °C) for 5 min. The RCR was calculated according to Formula (4). Taking the concentration of the sample solution as the horizontal coordinate and the clearance rate as the vertical coordinate, the linear equation between the sample solution and clearance rate was established to calculate the half-clearance concentration (EC_50_). Each sample was evaluated three times.

RCR (%) = ((A_0_ − A_1_)/A_0_) × 100% (4)

#### 2.5.3. Superoxygen (O^2−^) Free Radical Clearance

The superoxide free radical (O^2−^) clearance rate was measured according to Chi et al. [27] with minor modifications. A superoxide anion was generated by adding 0.1 mL 2.52 mmol/L nitrotetrazolium chloride blue (NBT), 0.1 mL 624 mmol/L reducing Coenzyme I disodium salt (NADH), and 0.1 mL peptide solution with different concentrations in a test tube. Then, 0.1 mL of 120 µmol/L phenazine N-methylsulfate (PMS) was added to each reaction mixture and, after incubating at 25 °C for 5 min, the absorbance was determined at 560 nm. The O^2−^ clearance rate (CRO) was calculated according to Equation (5), and each sample was evaluated three times. Taking the concentration of the sample solution as the horizontal coordinate and the clearance rate as the vertical coordinate, the linear equation between the sample solution and clearance rate was established to calculate the half-clearance concentration (EC_50_):

CRO (%) = ((A_0_ − A_1_)/A_0_) × 100% (5)

where A_1_ is the absorbance of the sample solution, and A_0_ is pure water instead of the absorbance of the sample solution.

#### 2.5.4. Hydroxyl (OH^−^·) Free Radical Clearance

The OH^−^· free radical clearance rate was determined according to You et al. [28] with slight modifications. Briefly, 0.15 mL 5 mmol/L 1, 10-phenanthroline, 0.15 mL 5 mmol/L FeSO_4_, 0.15 mL 15 mmol/L ethylenediamine tetraacetic acid (EDTA), and 0.1 mL 0.2 mmol/L phosphate buffer (pH 7.4) were mixed. Then, 0.15 mL peptide solution with different concentrations and 0.2 mL H_2_O_2_ (1%) were added. The mixture was incubated at 37 °C for 1 h and the absorbance was measured at 536 nm. The OH^−^· clearance rate (CROH) was calculated according to Formula (6) and the measurement was repeated three times for each sample. Taking the concentration of the sample solution as the horizontal coordinate and the clearance rate as the vertical coordinate, the linear equation between the sample solution and clearance rate was established to calculate the half-clearance concentration (EC_50_):

CROH (%) = (A_1_ − A_0_)/(A_2_ − A_0_) × 100% (6)

where A_1_ is the absorbance of the sample solution, A_2_ is the absorbance of the control solution without H_2_O_2_, and A_0_ is the absorbance of pure water.

### 2.6. Metal Ion Chelation

The Fe^2+^ chelating ability of the peptide was determined according to Nikoo et al. [29] with slight modifications. Here, 0.1 mL peptide solutions of different concentrations were mixed with 0.4 mL distilled water, and then 0.05 mL of 2 mmol/L FeCl_2_ solution and 0.1 mL of 5 mmol/L phenoxine solution were added, respectively, into each test tube. The absorbance was measured at 562 nm after the samples were incubated in a water bath at 25 °C for 10 min. The Fe^2+^ chelation rate (CRF) was calculated according to Formula (7), and each calculation was repeated three times. With the concentration of the sample solution as the horizontal coordinate and the Fe^2+^ chelation rate as the vertical coordinate, the linear equation of the chelation rate between the sample solution and Fe^2+^ was established, and the concentration of half of the chelation rate of Fe^2+^ IC_50_ was calculated:

CRF (%) = (1 − (A_1_ − A_2_)/A_0_) × 100% (7)

where A_1_ is the absorbance of the sample solution after reaction, A_2_ is the absorbance after pure water replaces FeCl_2_ solution in the reaction system, and A_0_ is the absorbance of pure water.

### 2.7. ACE-Inhibitory Activity Determination

The ACE-inhibitory activity of the peptide was determined according to the method described by Balti et al. [30] and Zhang et al. [31] with some alterations. The peptide sample was dissolved with 0.1 mol/L borate buffer (pH 8.3, containing 0.3 mol/L NaCl). Following this, 50 μL of 5 mmol/L hippuryl–histaminoyl–leucine (HHL) was added to 50 μL of peptide solution of different concentrations and incubated at 37 °C for 6 min. Then, 50 μL of 0.1 U/mL ACE solution was dissolved into 0.1 mol/L borate buffer (pH 8.3, containing 0.3 mol/L NaCl) to initiate the reaction. After further incubation at 37 °C for 30 min, 100 μL of 1 mol/L HCl was added to terminate the reaction. The content of hippuric acid released by ACE was determined with a K2025 HPLC (Shandong Wukong Instrument Co., Ltd., Jinan, China) at 228 nm. The chromatographic conditions were as follows: column, ODS-AP300A; mobile phase, acetonitrile (0.1%TFA)/water (0.1%TFA) = 25:75 (volume ratio), equal gradient elution; flow rate, 1 mL/min; temperature, 30 °C; sample size, 10 μL; detection wavelength, 228 nm. The ACE-inhibitory activity (ACEIA) was calculated in accordance with Equation (8) and the calculation was repeated three times for each sample. With the concentration of the sample solution as the horizontal coordinate and the ACE inhibition rate as the vertical coordinate, the linear equation between the sample solution and ACE inhibition rate was established to calculate the IC_50_ of the ACE inhibition rate:

ACEIA (%) = ((A_0_ − A_1_)/A_0_) × 100% (8)

where A_1_ is the peak area of the sample solution after reaction; and A_0_ is the peak area after the reaction of the control (0.1 mol/L borate buffer (pH 8.3, containing 0.3 mol/L NaCl) was used to replace the peptide sample).

### 2.8. Data Analysis

The data were processed using Microsoft Excel 2017 and expressed as the mean ± SD. Analysis of variance and significant differences (ANOVA) were performed on the data using IBM SPSS Statistics V24.0 (International Business Machines Corporation, New York, NY, USA), and the Fisher’s LSD test was used to evaluate significant differences between the data, where significance was set at *p* < 0.05.

## 3. Results and Analysis

### 3.1. Galactagogue Activity

The confirmation of the lactation-promoting effect of the white mullet meat can provide a natural and nutritious food choice for lactating women, help increase milk secretion, ensure adequate nutrition supply for infants, and improve their immunity and health level. In order to evaluate the milk secretion-promoting effect of the peptide obtained from white mullet meat via simulated digestion, animal experiments were carried out. With SD rats as the experimental objects, lactation volume, net weight gain, serum prolactin content, serum 5-HT content, and the mammary tissue of rats were measured, respectively.

#### 3.1.1. Effect of Peptide on Lactation Volume in Postpartum Rats

**Figure 1:**
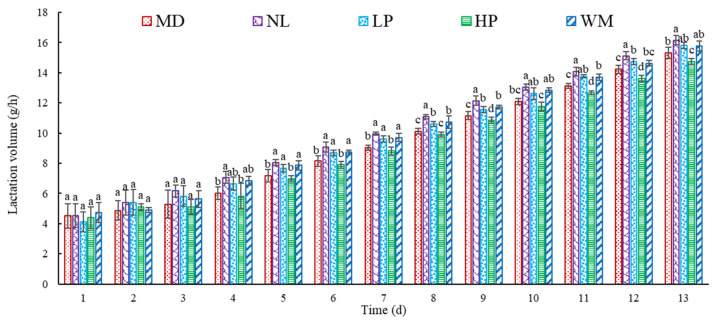
The lactation volume of the postpartum rats. MD, NL, LP, HP, and WM represent model group, normal group, low-dose peptide group, high-dose peptide group, and white mullet meat group, respectively. The lowercase letters on the bar represent significant (*p* < 0.05) differences within the group.

The lactation volume of the postpartum rats over 13 d is shown in Figure 1. The results indicated that the lactation volume of the postpartum rats in each group showed an increasing trend with the increase in the feeding time. In the first three days, there was no significant (*p* > 0.05) difference in the lactation volume in the model (MD), normal (NL), low-dose peptide (LP), high-dose peptide (HP), and white mullet (WM) groups. The lactation volume in the groups by the fourth day differed significantly (*p* < 0.05): the lactation volume in the NL group was significantly (*p* < 0.05) higher than that of the MD and HP groups, but not significantly (*p* < 0.05) higher than that of the LP and WM groups. Similar differences were observed on day 5, 6, 7, 10, and 13. The lactation volume for the NL group was significantly (*p* < 0.05) higher than that of the MD, LP, HP, and WM groups at day 8, 9, and 12. The lactation volume for the NL group was not significantly (*p* > 0.05) higher than that of the LP group at day 11, but it was markedly (*p* < 0.05) higher than that of the MD and HP groups. These results indicated that the lactation volume of the rats on the NL group was similar to that of those in the LP and WM groups during the measurement period, but was higher than that of the MD and HP rats, which suggests that the low-dose peptide and white mullet meat have the effect of inducing lactation in postpartum rats.

**Figure 2:**
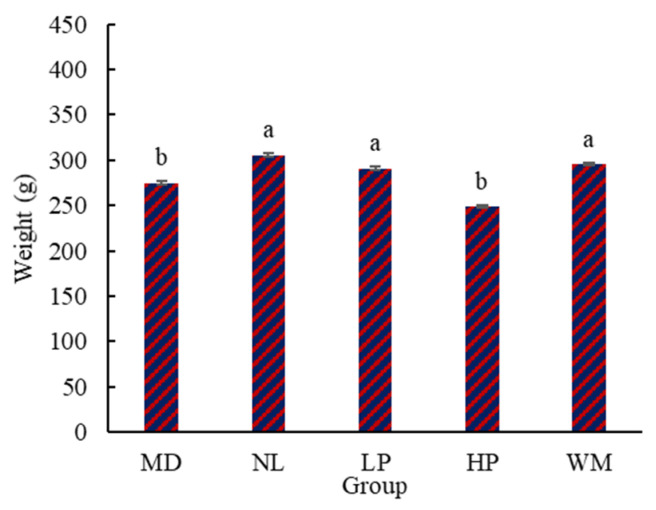
The net weight gain (NWG) of the rat pups. MD, NL, LP, HP, and WM represent model group, normal group, low-dose peptide group, high-dose peptide group, and white mullet meat group, respectively. The lowercase letters on the bar represent significant (*p* < 0.05) differences within the group.

Bromohintine is a substance obtained from ergot and can reduce milk secretion by inhibiting the secretion of prolactin in animals. The lactation volume of the rats in the NL group was higher than that of the MD rats from the second day, indicating that bromohintine is effective in inhibiting the secretion of milk in postpartum rats. The lactation volume of the rats in the WM group was higher than that of those in the MD group, supporting previous reports that white mullet meat has a milk secretion-promoting effect on postpartum women [4]. The lactation volume of the LP rats showed similar significant effects to those in the NL and WM groups, but the lactation volume of the LP rats was higher than that of the WM rats at day 11, 12, and 13. These results suggest that the peptide produced by the digestion of white mullet meat may have contributed more to the secretion of milk in the postpartum rats, further confirming that white mullet meat may promote the secretion of milk in postpartum women as well. The lactation volume of the HP rats was lower than that of the WM and LP rats, possibly due to a dose-dependent effect: higher amounts of peptide might induce digestive problems in rats [32], thus decreasing the promotion of milk secretion, but more investigation is needed to confirm this speculation. To further confirm the effect of the digested peptides and the white mullet meat on the secretion of milk in the postpartum rats, the net weight gain of the rat pups was determined (Figure 2).

#### 3.1.2. Effect of Peptide on NWG of Rat Pups

The data in Figure 2 show that the NWG of the rat pups in the NL, LP, and WM groups was markedly (*p* < 0.05) higher than that of those in the MD and HP groups. This result is consistent with the finding that the lactation volume of the NL, LP, and WM rats was higher than that of the MD and HP rats (Figure 1). The NWG of the HP rats was lower than that of the MD rats, but there was no marked (*p* > 0.05) difference. This result is similar to that of the lactation volume of the MD rats, which was higher than that of the HP rats (Figure 1).

NWG is an index to show the weight change of rat pups [25]: a higher value of MWG indicates that more breast milk was consumed and absorbed by the rat pups. Therefore, the results in Figure 2 further confirm that the white mullet meat and the low-dose digested peptides induced the secretion of milk in the postpartum rats, with the white mullet meat and its digested peptides having a milk secretion-promoting function.

#### 3.1.3. Content of Serum PRL in Rats

**Figure 3:**
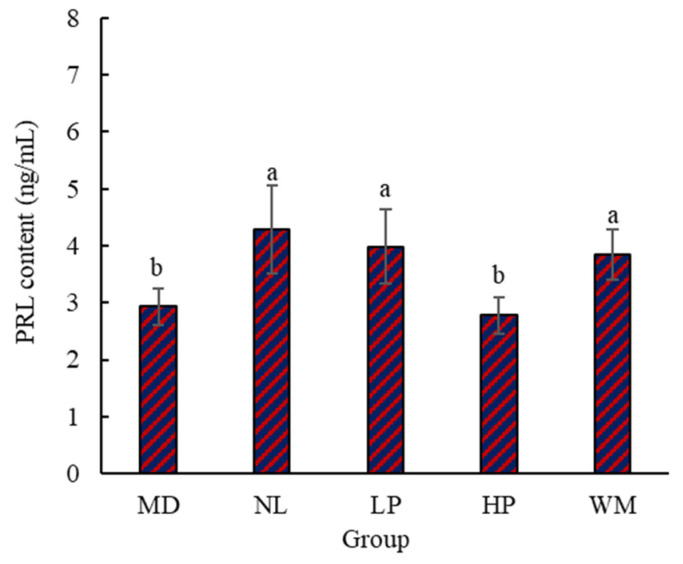
The content of PRL in the serum of the postpartum rats. MD, NL, LP, HP, and WM represent model group, normal group, low-dose peptide group, high-dose peptide group, and white mullet meat group, respectively. The lowercase letters on the bar represent significant (*p* < 0.05) differences within the group.

Lactation is a complex process affected by many factors not only related to the external environment, body, and mind, but also influenced by various hormones in the body [33]. Lactation is mainly caused by the action of various hormones on the developed mammary glands. Of these, PRL is a major hormone. Its main function is to promote the development and growth of mammary glands and stimulate and maintain lactation [34]. In order to further prove the effect of white mullet meat and its digested peptides on milk secretion in postpartum rats, the content of PRL in the serum of the postpartum rats was determined (Figure 3). The data in Figure 3 indicate that PRL levels in the NL, LP, and WM rats were significantly (*p* < 0.05) higher than those in the MD and HP rats. This result is consistent with the finding that the lactation volume of the NL, LP, and WM rats was higher than that of the MD and HP rats (Figure 1). The PRL level in the HP group was lower than that of the MD group, but with no significance (*p* > 0.05). This result is similar to the finding that the lactation volume of the MD rats was higher than that of the HP rats (Figure 1).

Bromocriptine can inhibit the production of milk in rats by inhibiting the secretion of PRL. This might be the reason why the PRL levels in the NL rats were markedly (*p* < 0.05) higher than those of the MD rats (Figure 3). The PRL levels of the NL, LP, and WM rats were not markedly (*p* > 0.05) different, but they were higher than that of the MD rats. This result suggests that white mullet meat and its digested peptides may promote milk secretion in postpartum rats through the interference or alleviation of the function of bromocriptine in rats. The PRL contents in the MD, NL, LP, HP, and WM rats were 2.93 ± 0.32 ng/mL, 4.28 ± 0.77 ng/mL, 3.99 ± 0.65 ng/mL, 2.78 ± 0.32 ng/mL, and 3.84 ± 0.44 ng/mL, respectively. The PRL content of the LP rats (3.99 ± 0.65 ng/mL) was higher (3.84 ± 0.32 ng/mL) than that of the WM rats. This result implies that digested peptides are more effective than white mullet meat in terms of the interference with, or alleviation of the function of bromocriptine in rats, which is further supported by the fact that the lactation volume of the LP rats was higher than that of the WM at day 13. As these peptides are obtained through the digestion of white mullet meat, it can be inferred that digested peptides are the main reason why the postpartum rats secreted more milk. This provides a basis for research into identifying and isolating the specific peptide that promotes milk secretion.

#### 3.1.4. Content of Serum 5-HT in Rats

**Figure 4:**
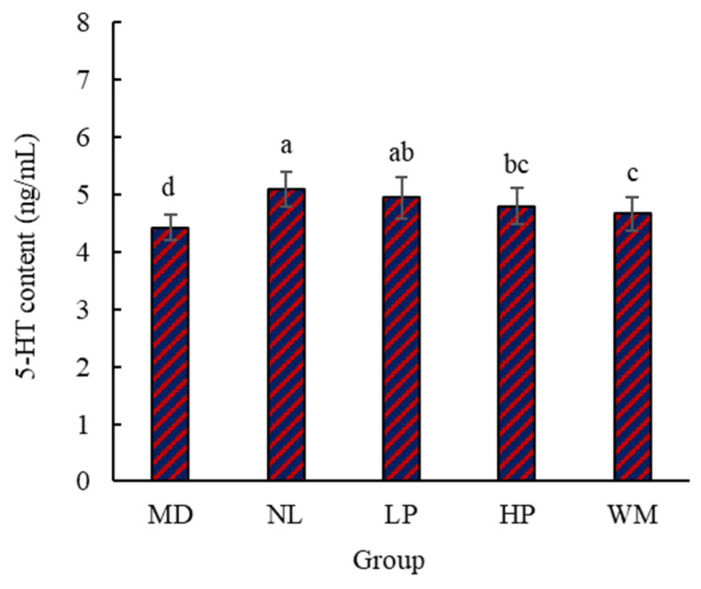
The content of serum 5-HT of the postpartum rats. MD, NL, LP, HP, and WM represent model group, normal group, low-dose peptide group, high-dose peptide group, and white mullet meat group, respectively. The lowercase letters on the bar represent significant (*p* < 0.05) differences within the group.

To further confirm the effect of white mullet meat and its digested peptides on the milk secretion of postpartum rats, the content of serum 5-HT was determined (Figure 4). The serum 5-HT contents for the MD, NL, LP, HP, and WM groups were 4.42 ng/mL, 5.10 ng/mL, 4.95 ng/mL, 4.80 ng/mL, and 4.66 ng/mL, respectively. The 5-HT content for the NL and LP groups was markedly (*p* < 0.05) higher than that of the MD, HP, and WM groups, with no marked (*p* > 0.05) difference in the 5-HT content for the NL and LP groups. This result further supports the assertion that digested peptides are the main reason for the greater milk secretion in the postpartum rats. The 5-HT content of the MD rats was the lowest.

5-HT is a neurotransmitter that plays a major role in regulating mood, sleep, appetite, etc. In addition, 5-HT can stimulate the release of PRL [35]. Therefore, the 5-HT content in serum should be similar to that of PRL in serum, as both can be used to explain the differences in milk secretion among the MD, NL, LP, HP, and WM groups. However, there was one difference: the 5-HT content for the WM rats was markedly (*p* < 0.05) lower than that of the NL rats, but the PRL content did not differ markedly (*p* > 0.05). The main cause for this difference is that the smell of the white mullet meat influenced the secretion of 5-HT. The feed used in the WM group contained 7% (*w*/*w*) white mullet meat, which has an obvious fishy smell [36]. This fishy smell may have decreased the appetite of the rats and thus reduced the secretion of 5-HT in the rats in the WM group.

#### 3.1.5. Histopathology of Rat Mammary Glands

**Figure 5:**
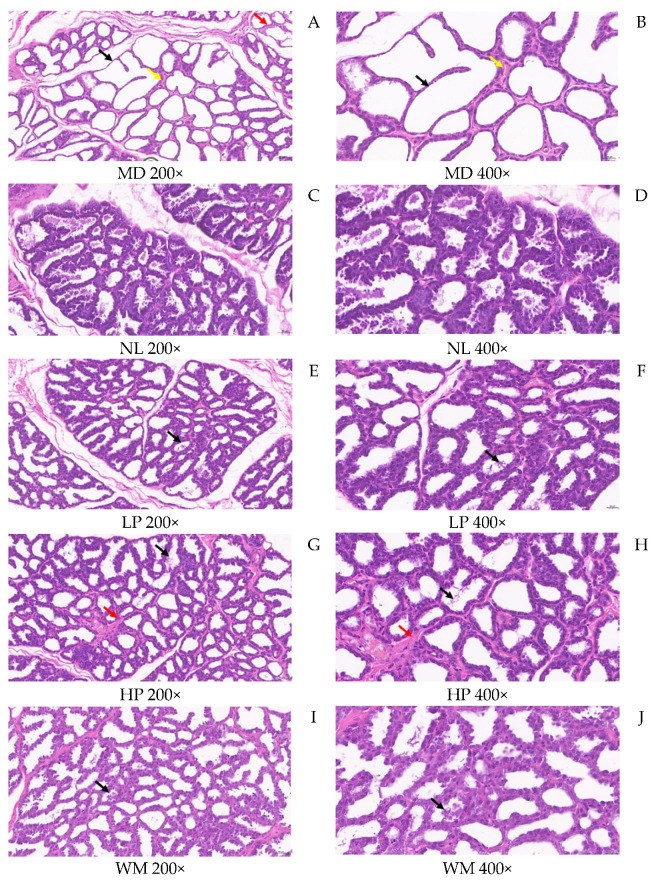
HE staining and histopathology of the postpartum rat gland. MD, NL, LP, HP, and WM represent model group, normal group, low-dose peptide group, high-dose peptide group, and white mullet meat group, respectively. 200× and 400× is magnification. (**A**) is a magnified 200× of MD; (**B**) is a magnified 400× of MD; (**C**) is a magnified 200× of NL; (**D**) is a magnified 400× of NL; (**E**) is a magnified 200× of LP; (**F**) is a magnified 400× of LP; (**G**) is a magnified 200× of HP; (**H**) is a magnified 400× of HP; (**I**) is a magnified 200× of WM; (**J**) is a magnified 400× of WM. Black arrow shows the acini severe dilation; red arrow shows flattened epithelium, significantly reduced secretion; yellow arrow shows mild interstitial hyperplasia. No significant abnormalities were observed in the normal group samples.

In order to evaluate the effect of white mullet meat and its digested peptides on the mammary gland tissue of rats, HE staining and histopathology were performed (Figure 5) according to the international standard of pathological changes in rats and mice [37]. Figure 5A,B show that the lesions in the MD rats (Figure 5A,B) are the most serious, with severe acinar expansion (black arrow), epithelial flatness, a large reduction in secretions (red arrow), and mild interstitial hyperplasia (yellow arrow). No obvious abnormalities were observed in the NL rats (Figure 5C,D). Compared with the MD rats (black arrow in Figure 5A,B), the LP (black arrow in Figure 5E,F), HP (black arrow in Figure 5G,H), and WM (black arrow in Figure 5I,J) rats showed fewer lesions and decreased secretions, and the HP rats showed mild interstitial hyperplasia (red arrow in Figure 5G,H).

Bromocriptine is a medicine that can be used to reduce the production and secretion of prolactin [38]. It is commonly used in experiments to verify the function of prolactin secretion-promoting target. Dong [39] used this method to study the therapeutic effect of prolactin decoction in a rat model of postpartum lactation deficiency. Therefore, the reduced number of lesions in the mammary gland tissue in rats might be another reason why milk secretion-promoting effects were observed in the LP and WM groups. This result further confirms that the milk secretion of LP and WM rats was higher than that of the MD rats. Low-dose peptide and white mullet can promote lactation in rats with lactation deficiency by reducing the number of lesions in the mammary gland tissue. This result also suggests that white mullet meat and its digested peptides have therapeutic effects on breast deficiency syndrome. This will be further investigated later.

### 3.2. Antioxidant Activity

**Figure 6:**
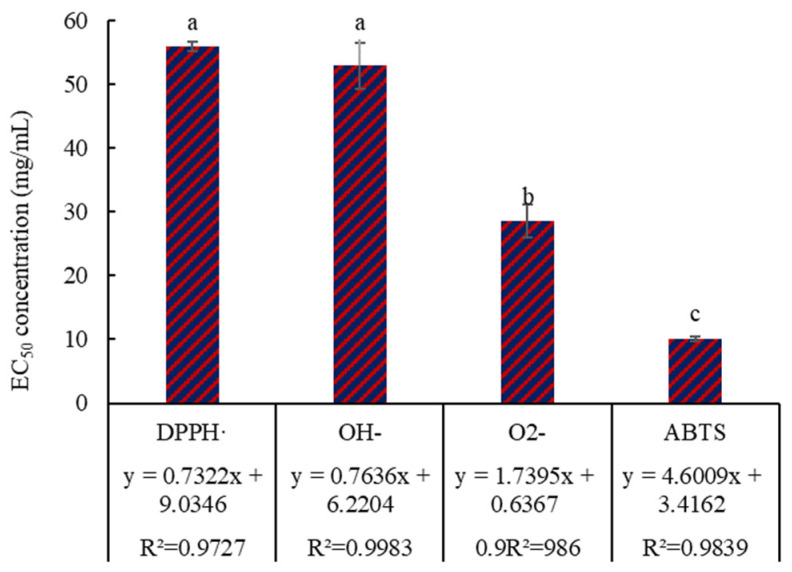
Antioxidant activity of the digested peptides. The lowercase letters on the bar indicate significant (*p* < 0.05) differences.

In order to evaluate the antioxidant activity of peptides prepared by simulated digestion, DPPH free radical scavenging rate, OH^−^ free radical scavenging rate, O^2−^ free radical scavenging rate, and ABTS free radical scavenging rate were measured for the prepared peptides (Figure 6). The results in Figure 6 indicate that the EC_50_ of the free radicals is significantly (*p* < 0.05) different, the EC_50_ of DPPH· and OH^−^ are markedly (*p* < 0.05) higher than that of O^2−^ and ABTS, there is no marked difference (*p* > 0.05) in the EC_50_ of DPPH· and OH^−^; and the EC_50_ of O^2−^ is markedly (*p* < 0.05) higher than that of ABTS. These results indicate that the digested peptides showed antioxidant activity. Given that a low EC_50_ value indicates a highly efficient free radical clearance ratio, it can be inferred that the clearance efficiency ranking of the digested peptides against free radicals is ABTS (10.14 mg/mL) > O^2−^ (28.53 mg/mL) > OH^−^ (52.92 mg/mL) > DPPH· (55.94 mg/mL).

Compared with the antioxidant activity of vitamin C (the half-clearance concentration of ABTS, O^2−^, OH^−^ and DPPH· is 0.080 mg/mL, 0.117 mg/mL, 0.361 mg/mL, and 0.093 mg/mL, respectively) [40,41], the antioxidant activity of digested peptides (the half-clearance concentration of ABTS, O^2−^, OH^−^, and DPPH· is 10.14 mg/mL, 28.53 mg/mL, 52.92 mg/mL, and 55.94 mg/mL) is weaker. Therefore, further purification might be necessary to improve the antioxidant activity of the digested peptides. DPPH· is a very stable nitrogen-centered free radical, and the three benzene rings in its structure provide a spatial barrier and contribute to its stability. This special molecular structure may be the reason why the EC_50_ of the DPPH· was significantly (*p* < 0.05) higher than that of O^2−^ and ABTS. OH^−^, O^2−^, and ABTS are obtained through chemical reactions, and the reaction speed may influence the EC_50_, thus resulting in significant differences (*p* < 0.05). There are also easily oxidized amino acid residues in the peptide, such as the thioether group in methionine, thiol group in cysteine, phenolic hydroxyl group in tyrosine, imidazole group in histidine, and indole group in tryptophan. These amino acid residues may contribute to the antioxidant activity of the digested peptides.

### 3.3. ACE-Inhibitory Activity

**Figure 7:**
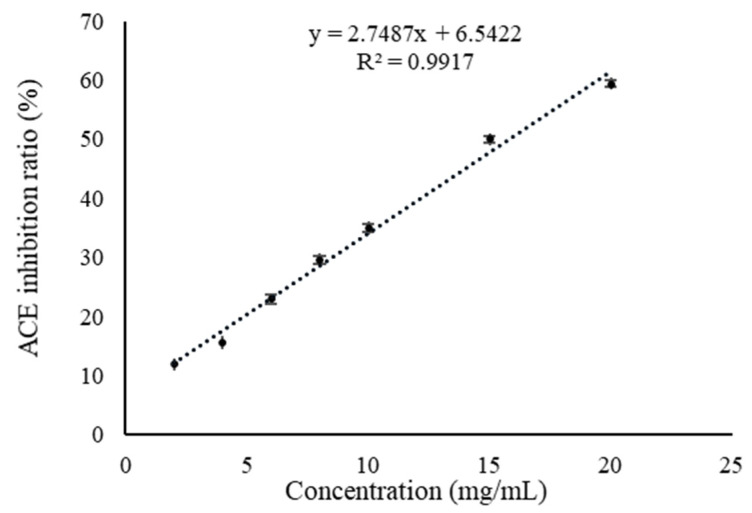
ACE inhibition ratio of the digested peptides.

ACE plays an important role in regulating blood pressure; it not only inactivates vasodilator bradykinin, but also promotes the conversion of angiotensin I to angiotensin II, leading to hypertension [42]. To evaluate the effect of the digested peptides on hypertension, the ACE inhibition activity was determined (Figure 7). The data in Figure 7 indicate that there is a highly linear correlation (y = 2.7487x + 6.5422, R^2^ = 0.9917) between the ACE inhibition ratio and the concentration of digested peptides. The IC_50_ of the digested peptides to ACE inhibition ratio is 15.81 mg/mL, indicating ACE inhibition activity.

The IC_50_ of the digested peptides to ACE inhibition ratio (15.81 mg/mL) is higher than that of ACE inhibitor drug captopril (5.43 × 10^−5^ mg/mL), indicating the ACE inhibitory activity of the digested peptides is weaker than that of antihypertensive drug. Therefore, the digested peptides might be suitable for assisting in regulating hypertension and not suitable for treating it. ACE has a higher affinity for substrates or competitive inhibitors containing residues of aromatic amino acids (Trp, Phe, Tyr, Pro) and branched aliphatic hydrophobic amino acids (Gly, Val, Leu, Ile) [31]. In addition, the presence of positively charged amino acids (Lys, His, Arg) in the peptide sequence also contributed to the enhancement of ACE-inhibitory activity. The fact that the digested peptides contain these amino acid residues could be the main reason for its ACE-inhibitory activity.

### 3.4. Fe^2+^ Chelation

**Figure 8:**
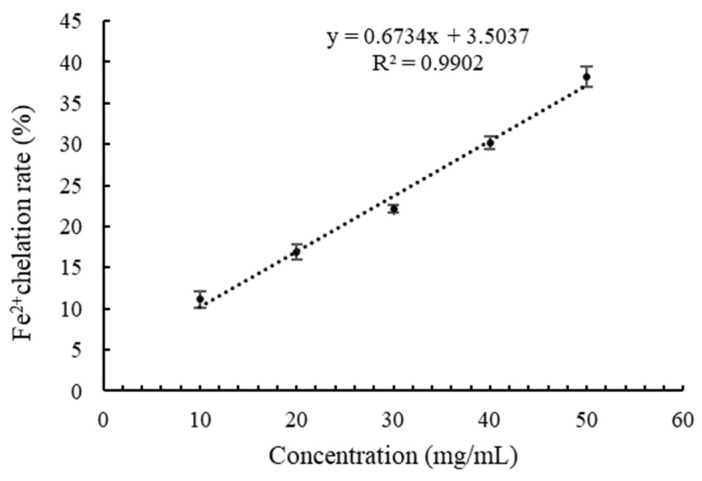
Fe^2+^ chelation ratio of the digested peptides.

Dietary minerals have a significant impact on human health. For example, iron deficiency may lead to anemia and could damage hematopoietic function in the body. However, dietary supplements alone may not be enough to meet human physiological needs, meaning that additional mineral supplements are required [43]. To analyze the effect of the digested peptides on promoting the absorption of iron elements, its Fe^2+^ chelation effect was determined (Figure 8). Figure 8 shows that the Fe^2+^ chelation ratio was linearly correlated (y = 0.6734x + 3.5037, R^2^ = 0.9902) with the concentration of digested peptides. The IC_50_ of the digested peptides to Fe^2+^ chelation was 69.05 mg/mL, indicating Fe^2+^ chelation activity.

The IC_50_ of the digested peptides to Fe^2+^ chelation (69.05 mg/mL) is higher than that of ethylene diamine tetraacetic acid (EDTA), whose IC_50_ to Fe^2+^ chelation is 0.146 μg/mL under the conditions of an initial concentration of 1 μM Fe^2+^ and pH 7. This result indicates that the Fe^2+^ chelation activity of the digested peptide is weaker than EDTA, suggesting further purification might be necessary to improve the chelating ability of the digested peptides towards Fe^2+^. Studies have shown that acidic or basic amino acid residues contained in peptides can exhibit chelation effects on ferrous ions [44]. Amino acid residues belong to carboxyl, amino, and guanidine groups and can combine with ferrous ions through electrostatic interaction or coordination [42]. Some amino acid residues can chelate with ferrous ions, which may explain why the digested peptides show Fe^2+^ chelation activity.

## 4. Conclusions

White mullet meat was hydrolyzed using simulated digestion in vitro, and the functions of the obtained peptide were evaluated. The results indicated that the peptide had milk secretion-promoting, antioxidant, ACE-inhibitory, and Fe^2+^ chelation abilities. This conclusion is expected to provide a reference for further study on the functional activities of white mullet meat and its hydrolyzed peptides. Although this investigation has confirmed that the digestive peptides of the white mullet meat have milk secretion-promoting, antioxidant, ACE inhibitory, and Fe^2+^ chelation abilities, this study did not investigate the specific peptides that produce these effects, and did not compare their functionality with existing functional chemicals. Therefore, these contents will be given priority in future research.

## Data Availability

The original contributions presented in this study are included in the article. Further inquiries can be directed to the corresponding author.
